# Riding the storm: managing cytokine-related toxicities in CAR-T cell therapy

**DOI:** 10.1007/s00281-024-01013-w

**Published:** 2024-07-16

**Authors:** Andrew D. Hughes, David T. Teachey, Caroline Diorio

**Affiliations:** 1 Division of Oncology, Department of Pediatrics, Children’s Hospital of Philadelphia, University of Pennsylvania Perelman School of Medicine, Philadelphia, PA USA; 2 Immune Dysregulation Frontier Program, Department of Pediatrics, Children’s Hospital of Philadelphia, University of Pennsylvania Perelman School of Medicine, Philadelphia, PA USA

**Keywords:** Chimeric antigen receptor T cells (CAR-T), CAR-T toxicity, cytokine release syndrome (CRS), immune effector cell-associated neurotoxicity (ICANS), immune effector cell-assocaited HLH-like syndrome (IEC-HS)

## Abstract

The advent of chimeric antigen receptor T cells (CAR-T) has been a paradigm shift in cancer immunotherapeutics, with remarkable outcomes reported for a growing catalog of malignancies. While CAR-T are highly effective in multiple diseases, salvaging patients who were considered incurable, they have unique toxicities which can be life-threatening. Understanding the biology and risk factors for these toxicities has led to targeted treatment approaches which can mitigate them successfully. The three toxicities of particular interest are cytokine release syndrome (CRS), immune effector cell-associated neurotoxicity syndrome (ICANS), and immune effector cell-associated hemophagocytic lymphohistiocytosis (HLH)-like syndrome (IEC-HS). Each of these is characterized by cytokine storm and hyperinflammation; however, they differ mechanistically with regard to the cytokines and immune cells that drive the pathophysiology. We summarize the current state of the field of CAR-T-associated toxicities, focusing on underlying biology and how this informs toxicity management and prevention. We also highlight several emerging agents showing promise in preclinical models and the clinic. Many of these established and emerging agents do not appear to impact the anti-tumor function of CAR-T, opening the door to additional and wider CAR-T applications.

## Introduction

The earliest clinical reports of chimeric antigen receptor T cells (CAR-T) described a syndrome of fevers, sometimes accompanied by hypoxia, hypotension, and cytopenias, termed cytokine release syndrome (CRS). The first patient who had sustained remission after CAR-T experienced a syndrome of fever, chills, and fatigue beginning two days after infusion and peaking over the subsequent 5 days, conincident with CAR-T cell expansion [1]. A subsequent report described 10 patients with relapsed/refractory B cell leukemias infused with CD19-directed CAR-T, some of the first patients to be treated with this novel therapy: 7 of 9 patients had fever, 3 developed hypotension, and one patient died of a sepsis-like picture with hypotension and renal failure [2]. The first pediatric patient to receive CAR-T received a CD19-directed product for treatment of B cell acute lymphoblastic lymphoma (B-ALL), and developed a syndrome of high-spiking fevers with progression to respiratory and cardiovascular failure, requiring mechanical ventilation and multiple vasopressors [3]. This patient had numerous cytokine elevations, including interleukin-6 (IL-6), interferon-gamma (IFNγ), and IL-10, and clinically did not respond significantly to steroids or etanercept but had a dramatic improvement after she received tocilizumab and remains disease-free now 10 years after infusion. Despite these significant toxicities, CAR-T products have achieved high complete response rates in multiple types of cancer including several pediatric and adult hematologic malignancies [4].

Although the advent of CAR-T therapy has marked a revolution in the treatment of certain cancers, it has also brought unique and serious toxicity profiles, distinct from toxicities seen with traditional cytotoxic therapies. CRS is now recognized as the most common potentially life-threatening CAR-T toxicity and is broadly characterized by inflammation resulting in fever and capillary leak leading to hypotension and hypoxia. The second most common potentially life-threatening toxicity, immune effector cell-associated neurotoxicity syndrome (ICANS), is marked by confusion, ataxia, cranial nerve palsies, and seizure and is hypothesized to be related to cytokine dysregulation in the CNS [5]. An emergent CAR-T toxicity, termed immune effector cell-associated hemophagocytic lymphohistiocytosis (HLH)-like syndrome (IEC-HS), is typified by late onset hyperinflammation with high ferritin, coagulopathy, cytopenias, and multiorgan failure [6]. IEC-HS has only recently been recognized as a distinct entity to underscore its independence from previously described syndromes, requiring discrete diagnostic and management guidelines [6]. These toxicities are due in large part to cytokine dysregulation occurring following CAR-T. They can all occur to varying degrees in other immune effector cell (IEC) therapies, including bispecific T-cell engagers (BiTE) which target endogenous T cells to cancer cells. There is also a growing catalog of adoptive cell therapies utilizing non-T cell effectors, for example NK cells, which may ultimately be found to create their own patterns of toxicities based on their different cellular biology.

The aim of this manuscript is to summarize the current understanding of the biology of these toxicities, the most up to date and evidence-based consensus guidelines for their management, highlight emerging management options, and identify gaps in our understanding of CAR-T mediated toxicities. A few of these emerging toxicity-mitigating therapies have the potential to revolutionize outcomes with CAR-T therapy, paving the way for further CAR-T development and application. This will also lay the groundwork for readers to understand the current major unanswered questions in the field (Box 1).

**Box 1:** Ongoing debates and questions surrounding CAR-T-related toxicities

• Reports are conflicting regarding the effect of corticosteroids (CS) on CAR-T anti-tumor function. While it has been demonstrated in several cases that CS are safe and effective, concern remains that CS will ultimately be harmful to long term efficacy. • CS continue as the first-line therapy recommendation for ICANS, however other more targeted agents may be of benefit and are an important are of current study. • Early intervention is proving to be effective in prevention of more severe cases of CRS and ICANS, and preemptive strategies are being explored as well. • As more targeted strategies for specific types of CAR-T-related toxicities prove to be effective, how specific will toxicity mitigation become? It is ossible that different CAR-T constructs, and different types of IEC therapy in general, will be best managed by different approaches. • A nascent field is CAR-T for solid tumors. It will be interesting to see what toxicities are seen in these applications and it may not be the case that the current guidelines for CRS, ICANS, and IEC-HS generated from experience with CAR-T for hematologic malignancies will be as effective.	

## Cytokine release syndrome

The American Society for Transplantation and Cellular Therapy (ASTCT) recently convened a panel of experts from a variety of disciplines to create a unifying definition and grading system for CRS and ICANS [7]. Prior to this meeting, there were a handful of disparate definitions and grading systems that were developed as the field of CAR-T-related toxicity has matured, and the lack of uniformity created problems in comparing outcomes and toxicities between studies. CRS is currently defined as “a supraphysiologic response following any immune therapy that results in the activation or engagement of endogenous or infused T cells and/or other immune effector cells. Symptoms can be progressive, must include fever at the onset, and may include hypotension, capillary leak (hypoxia) and end organ dysfunction” [7].

### CRS grading

The grading scheme devised in the ASTCT consensus statement inclues fever as a necessary feature of all grades of CRS, and the maximum severity of hypotension or hypoxia defines the grade [7, 8].

- Grade 1: fever without hypotension or hypoxia, can be associated with constitutional symptoms such as myalgia and malaise.
- Grade 2: fever with hypotension and/or hypoxia requiring minimal support, such as fluids and low-flow nasal cannula, respectively.
- Grade 3: hypotension requiring one vasopressor and/or respiratory distress requiring high-flow nasal cannula or facemask.
- Grade 4: hypotention requiring more than one vasopressor (excluding vasopressin) and/or hypoxia requiring positive pressure ventilation including intubation.
- Grade 5: death.

### CRS incidence

The incidence of CRS is impacted by a number of important variables, including the co-stimulatory domain used in the CAR construct, the target antigen of the CAR, *ex vivo* processing/culturing parameters, *ex vivo* T cell selection processes, and the biology of the target tumor itself including tumor burden. Currently, the most widely used CAR-T products are tisagenlecleucel (tisa-cel), axicabtagene ciloleucel (axi-cel), and brexucabtagene autleucel (KTE-X19), all CD19-targeted products that differ in their costimulatory domain with tisa-cel using 4-1BB and axi-cel and KTE-X19 using CD28 [9, 10]. The two landmark trials of CAR-T for relapsed/refractory large B cell lymphoma, ZUMA-1 and JULIET, treated patients with axi-cel and tisa-cel, respectively, and found different frequencies of CRS: on ZUMA-1 93% of patients had any grade of CRS with 13% of patients experiencing severe (grade >=3) CRS, while on JULIET 58% of patients had any grade of CRS with 22% of patients having severe symptoms [11, 12]. The numbers of patients treated for CRS also differed, with 43% of patients receiving IL-6 blockade and 27% receiving steroids on ZUMA-1 compared to 14% and 10%, respectively, on JULIET. The trials ZUMA-2 and ZUMA-3 tested KTE-X19 for relapsed/refractory (r/r) mantle cell lymphoma and r/r adult B-ALL, respectively, and found similar rates of CRS (91% and 89% with any grade CRS, 15% and 24% of patients with severe CRS, respectively) [13, 14]. In a more recent large adult cohort comparing axi-cel and tisa-cel for the treatment of diffuse large B cell lymphoma (DLBCL), CRS occurred in 86% of patients treated with axi-cel (6% of those being severe) versus 76% of patients treated with tisa-cel (12% of those being severe) [15]. Conversely, for non-Hodgkins lymphoma treated with axi-cel on the ZUMA-5 trial, rates were much lower with only 9% of patients experiencing grade >=3 CRS [16]. Practices for what interventions to take for CAR-T related toxicities, when to act, and ways to modulate disease-specific characteristics (e.g. reduction in tumor burden) have changed over time and have likely influenced toxicity incidence.

### CRS biology and markers

**Fig. 1:**
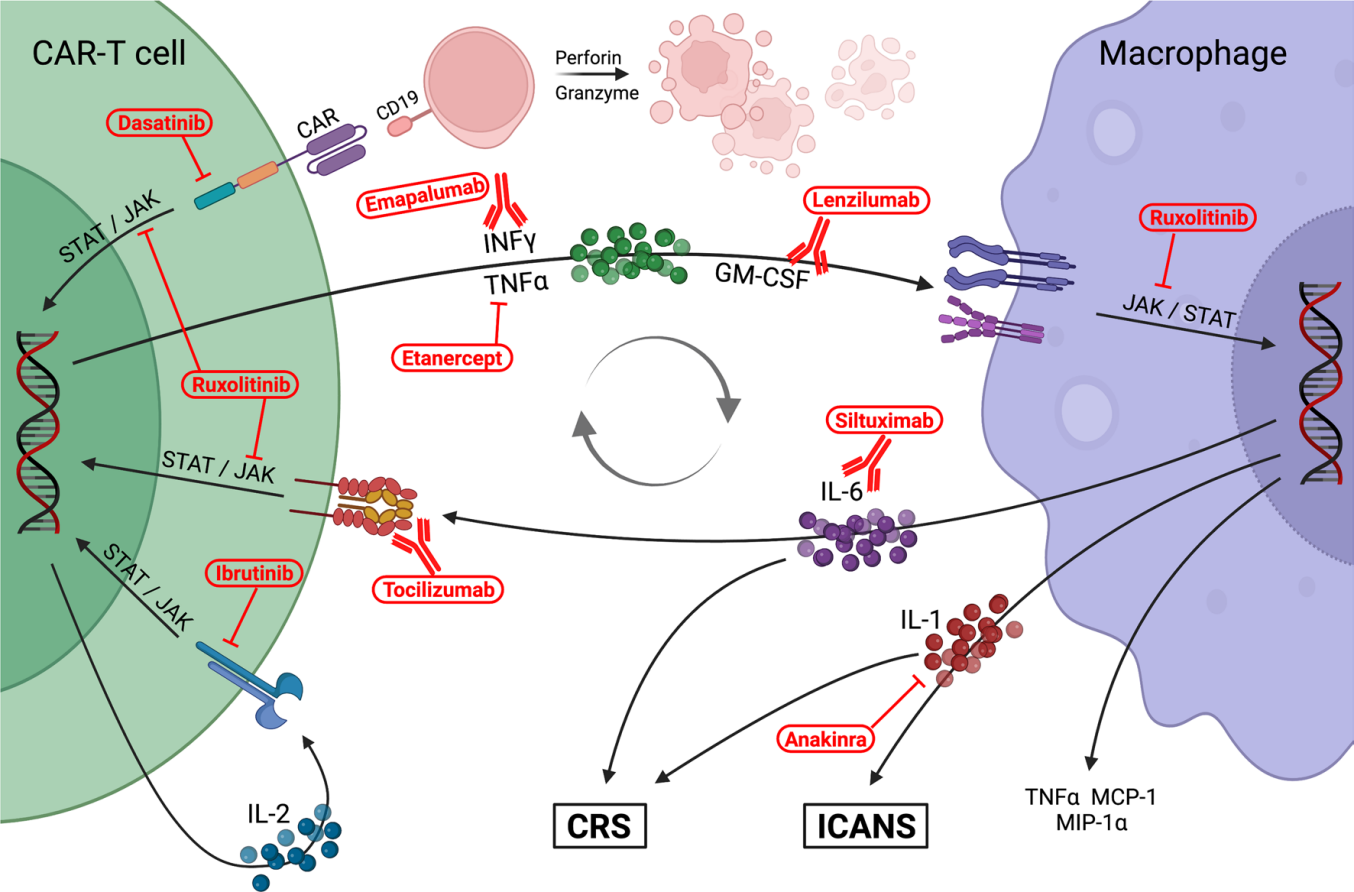
Schematic detailing the current understanding of the pathophysiology of CRS and ICANS caused by cross-talk between CAR-T cells and macrophages. Several of the current and emerging therapies for CRS and ICANS are shown in their mechanism of action. Created with biorender.com

The current understanding of CRS is that CAR-T engagement leads to proliferation of T cells and secretion of pro-inflammatory cytokines that futher activate CAR-T as well as the host immune system, causing symptoms such as fever and off-target tissue damage. Extensive studies have measured the cytokine profiles of CRS with CAR-T and have identified the signaling pathways that are dysregulated in this clinical syndrome (Fig. 1). The importance of IL-6 in CRS is well established [17–21], and IFNγ is increasingly being recognized as a key cytokine in not just CRS but other CAR-T mediated toxicities [20, 22–26]. In general, cytokines released by activated T cells are elevated in CRS, specifically IL-6, IFNγ, soluble IL-2 receptor (sIL-2R), sIL-2Ra, and granulocyte-monocyte colony stimulating factor (GM-CSF). Activated monocytes/macrophages secrete high levels of cytokines in turn, including IL-1Ra, IL-10, IL-6, CXC motif chemokine ligand 9/monokine induced by gamma (CXCL9; MIG), CXCL10 (IP-10), IFNa, macrophage-inflammatory protein-1 alpha (MIP1a), MIP1b, and sIL-6R [5, 27].

In comparing patients with mild (grade 0-3) CRS to those with severe (grade 4-5) CRS in patients treated with a 4-1BB CD19-directed CAR-T product, Teachey et al. found IFNγ, IL-6, IL-8, sIL-2R, sgp130, sIL-6R, MCP1, MIP1α, MIP1β, and GM-CSF to be significantly associated with the more severe grades [20]. Similarly, ferritin and C-reactive protein (CRP) were significantly higher in severe CRS, and notably ferritin was >10,000 mg/dL in every patient with severe CRS. Hypofibrinogenemia was also found in patients with severe CRS. This laboratory profile is nearly identical to that seen in patients with macrophage activation syndrome (MAS)/HLH, for which a value of ferritin >10,000 is considered sensitive and specific, drawing a striking parallel between the two syndromes [20].

An important concept of CAR-T therapy is that many of the pathways that lead to toxicities are not the same as those involved in target cell killing. This is apparent in the finding that the development of toxicity does not correlate with clinical response; while both are outcomes of CAR-T cell activity, they are not mutually necessary. For example, a retrospective analysis of hundreds of adults treated with axi-cel or tisa-cel compared those who developed CRS with those who did not and found no difference in progression free survival (P=0.99) or overall survival (P=0.16) [28].

### CRS predictors

Several factors have been found to be predictive of CRS severity, and can broadly be categorized into disease characteristics, therapy characteristics, and laboratory parameters. Disease burden has long been identified as being correlated with severity of toxicity, with higher tumor burden and bulky disease being associated with worse symptoms [26, 29–32]. In one study of pediatric patients with B-ALL receiving CAR-T, low disease burden had a strong negative predictive value (1 of 15 patients with <5% blasts in bone marrow developed severe CRS), however, disease burden alone was not sufficient to predict which patients will develop severe CRS (10 of 23 patients with >25% disease in bone marrow developed severe CRS) [20]. Highly proliferative malignancies such as B-ALL and DLBCL are associated with more severe CRS when compared to less active diseases including follicular lymphoma and myeloma. However, calculating the growth rate of lymphomas prior to CAR-T infusion was found to be not predictive of toxicity [33], implying that high proliferation is not necessarily associated with a higher risk of CRS [34]. Age within adulthood has not been found to be associated with toxicity rates [35–37].

Higher CAR-T doses are predictive of worse symptoms [38], and specific costimulatory domains, such as CD28 (eg. axi-cel), have higher rates of CRS and neurotoxicity than those with 4-1BB (eg. tisa-cel) [15]. The CAR itself can play a role in the development of CRS based on its molecular characteristics. For example, manipulating the structure of CD8α domain of the CAR, while keeping the 4-1BB/CD28 and CD3ζ signaling domains unchanged was found to preserve anti-tumor efficacy in patients with B cell lymphoma while significantly reducing the incidence CRS and neurotoxicity [39, 40]. Separately, lowering the affinity of the antibody-derived domain of the CAR resulted in significantly lower rates of CRS and neurotoxicity with preserved clinical efficacy in a small cohort of pediatric patients with B-ALL [41]. The CAR-T target also plays a role in toxicity profiles based on on-target off-tumor effects. For example, neurotoxicity may be seen more frequently with CD19-directed agents due to the presence of CD19 on mural cells in the brain [42], whereas the unique movement-related toxicities seen with BCMA-directed CAR-T products and may be attributable to BCMA expression in separate areas of the CNS [43].

While cytokine levels have not been found to correlate with CRS severity [44], a number of schema for predicting which patients will develop severe or non-severe CRS have been developed based on retrospective analyses [17, 45, 46]. A recent proteomic analysis identified elevations in MILR1, a negative regulator of hypersensitivity reactions, to be predictive of CRS with high sensitivity and sepecificity (97, 88%) [20]. Pennisi et al. modified the Endothelial Activation and Stress Index (EASIX) score, used to predict outcomes in HSCT, by replacing creatinine with CRP to create the formula lactate dehydrogenase (LDH) x CRP/platelet count and found good association between development of severe CRS and ICANS and this modified score [47]. In a unique approach, Jalota et al explored pretreatment metabolic parameters and found that higher glucose, lower glutamine, and lower cholesterol were associated with earlier onset CRS [48]. While several of these boast remarkable specificities/sensitivities, their utility in prospective risk stratification is yet to be determined.

### CRS therapies

While it is clearly established that cytokines play a role in CRS, cytokines are also necessary for T cell function and proliferation, and so the effect on the anti-cancer action of T cells by cytokine blockade has been a major concern. Fortunately, several agents have been found to have mitigating effects on cytokine-related toxicity (Table 1) without dampening cancer cell killing. The IL-6 receptor antagonist tocilizumab is a highly effective and the longest-used treatment for CRS [49], and has been shown to have no effect on CAR-T cell expansion [51]. Notably, tocilizumab administration has not been associated with any major adverse reactions of its own [49]. Tocilizumab is therefore firmly established as the first-line treatment for CRS [8] and can be redosed at 8-hour intervals. Siltuximab, a monoclonal antibody specific to IL-6, has been shown to have similar efficacy to tocilizumab; however, they have not been compared directly in any published trials [60, 73].

**Table 1:** Established and emerging therapies for CRS

	Agent	Rationale	Comments	Notable references
First line	Tocilizumab	IL-6 is released by macrophages in CRS	Well established as frontline treatment for CRS. Early treatment shown to be more effective than late. Can repeat doses q8h if insufficient response to first dose.	[8, 49, 50]
Second line	Corticosteroids	CS achieve broad immunosuppression	Evidence mounting that steroids do not impair CAR-T efficacy, but conflicting reports remain. Methylprednisolone preferred for more severe CRS, dexamethasone preferred with concomitant ICANS. Earlier start of CS associated with lower cumulative doses required.	[8, 51–53]
Third line	Anakinra	IL-1 found to play a primary role in mediating CRS	Increasing use for all CAR-T-related toxicities. Attractive safety profile. Dose can be modulated to effect, which allows for weaning off with recovery.	[54–56]
Additional strategies	Safety switch	Engineered mechanism to disable CAR-T and abolish the source of inflammation	Multiple mechanisms in development or reported, both reversible and irreversible varieties.	[57]
	Emapalumab	IFNγ shown to mediate CRS/ICANS in preclinical model	Successfully used in small numbers of patients with refractory CRS	[58, 59]
	Siltuximab	Role of IL-6 well established in CRS	Scattered reports of use for higher grade CRS refractory to tocilizumab	[60]
	Dasatinib	Tyrosine kinase inhibitor that blocks signal transduction through T cell receptor, shown in preclinical study to suppress CAR-T cell activation	Clinical trial currently accruing (NCT04603872) testing dasatinib combined with CART therapy. Also being studied as an agent to "rest" CAR-T to reverse exhaustion.	[61–63]
	Ibrutinib	Tyrosine kinase inhibitor that blocks IL-2 signaling, reduces cytokine release by T cells	Concurrent administration of ibrutinib with CD19 CAR-T in small number of patients with CLL resulted in lower CRS severity without statistical difference in CART expansion or disease control.	[64, 65]
	Ruxolitinib	JAK/STAT mediates signaling by several pro-inflammatory cytokines important in CRS	Case reports have demonstrated activity of ruxolitinib in refractory CRS	[66, 67]
	Etanercept	TNFα elevated during CRS	Since case report of treatment of CRS after BCMA CART showing efficacy and no impedance of CART activity	[68]
Preemptive therapy	Tocilizumab	Earlier tocilizumab administration results in less severe CRS, therefore pre-emptive treatment may have an even greater effect	Single arm trial administered tocilizumab with onset of grade 1 CRS for patients with higher tumor burden getting CTL019 and found near 50% reduction in severe CRS without impacting efficacy or CAR-T persistence compared to historical controls	[51, 69, 70]
	Anakinra	Preclinical model demonstrated that IL-1 inhibition prevented severe CRS and ICANS	Clinical trial currently underway (NCT04148430) studying anakinra for prevention of CRS and ICANS in adults receiving CD19-directed CAR-T	[55, 71]
	Lenzilumab	GM-CSF elevations found to correlate with severe CRS and neurotoxicity on ZUMA-1	Administration before CAR-T infusion in a small number of patients resulted in very low rates of CRS and ICANS	[72]

As tocilizumab has been found to both be a potent treatment for CRS and not impair the function of CAR-T cells, it follows that tocilizumab could be used to prevent CRS rather than treat it. A few trials have investigated using tocilizumab as a preemptive measure, initiating treatment at low-grade toxicity, and found that this strategy was effective in prevening the onset of severe CRS [51, 69, 70]. Separately, in one single-arm study 20 patients were given tocilizumab prior to CAR-T infusion and reported that there were no cases of severe CRS (grade 3 or higher), and while 5 patients had ICANS, 4 of these were grade 1 [74].

Corticosteroids (CS) are currently recommended as second-line therapy for CRS that persists or progresses after tocilizumab [8]. There has long been concern for CS having deleterious effect on CAR-T function and persistence. Some studies have suggested that CS are disadvantageous for CAR-T cell function [21, 75], however others have not identified any short-term deleterious impact from using CS [51, 52, 76]. Ultimately, it is likely that the effect of CS is a dose-dependent, and short-term use of steroids, even at high dose, were found in retrospective analysis to not affect outcomes of CAR-T therapy while cumulative doses were associated with shorter survival [75]. Earlier administration of CS is associated with shorter courses of CS being used [53], and early CS administration was found to prevent progression to severe CRS on the ZUMA-1 trial [76].

The agent with the most published clinical experience for refractory CRS is likely the IL-1 receptor antagonist anakinra. Preclinical models of CAR-T-derived CRS have found that monocyte/macrophage-derived IL-1 and IL-6 are both important in the pathophysiology of CRS and ICANS and that blockade of IL-1 with anakinra alone is effective in mitigating CRS and ICANS [54, 77]. Clinical data supporting the use of anakinra for severe CRS is growing, and prospective studies are underway for use of anakinra for treatment and prevention of CRS and ICANS [55]. Importantly anakinra does not appear to impact CAR-T efficacy [77, 78].

Several emerging therapies have shown promise for the treatment of CRS (Table 1), to date only reported in small numbers of patients. Based on the finding that IFNγ is elevated in CRS, and is a major avenue for activation of macrophages by T cells, the IFNγ-blocking antibody emapalumab has been utilized successfully for severe CRS [58, 79], and promisingly IFNγ blockade has not been found to compromise CAR-T function *in vitro* and *in vivo* in xenograft models of hematologic malignancies [59], although this has found to not hold true for solid tumors [46]. Ruxolitinib has been used in some patients [66], operating on the concept that many inflammatory cytokines signal through JAK/STAT pathways [80]. In one report of 4 patients, ruxolitinib resulted in rapid resolution of CRS symptoms and decrease in serum cytokines associated with CRS [67]. Both emapalumab and ruxolitinib are relatively well tolerated. Ruxolitinib can cause cytopenias [81, 82], and both agents increased the risk of viral reactivations [83, 84]. Another drawback of emapalumab is that it is quite expensive, however 1-2 doses have been sufficient in amelioriating severe CRS in published cases [24, 58].

TNFα blockade with etanercept was reported in three patients treated with BCMA-directed CAR-T [68]. The patients had good therapeutic response, and the authors note no apparent impact on CAR-T efficacy. Etanercept has been previously used with unclear efficacy in CRS related to other CAR-T [3]. GM-CSF levels were found to correlate with severe CRS and neurotoxicity on ZUMA-7 [85], which has sparked interest in the monoclonal antibody against GM-CSF, lenzilumab, for CRS and ICANS. In a preclinical model lenzilumab was found to suppress CAR-T toxicity without decreasing efficacy [86]. In a preventative approach, the single-arm phase 1 ZUMA-19 trial administered lenzilumab prior to axi-cel infusion for 6 adults with (r/r) large B cell lymphoma (LBCL) and no patient developed severe CRS and one developed grade>=3 neurotoxicity with no dose-limiting toxicities attributable to lenzilumab [72].

Also of interest are tyrosine kinase inhibitors which interrupt signal transduction through activating receptors including the CAR. For example, dasatinib has been shown to inhibit phosphorylation of CD3ζ and ZAP70, which blocks signaling through the CAR. This can be used to reversibly tune CAR signaling, and while it does reduce the anti-tumor effect of CAR-T its overall activity has been found to mitigate toxicity and improve CAR-T persistence [61–63]. Separately, the Bruton’s tyrosine kinase inhibitor ibrutinib, which inhibits IL-2 signaling, was administered concurrently with CD19-directed CAR-T in 19 patients and resulted in low CRS severity and high rates of disease response [64].

Certain CAR-T are being designed with so-called safety switches, or molecular mechanisms by which the CAR-T can be “turned off,” halting unacceptable toxicities. One approach incorporates an inducible caspase 9 fusion protein into CAR-T cells that dimerizes on exposure to the synthetic drug rimiducid, inducing apoptosis [87]. Numerous novel CAR-T products have been created more recently that incorporate different off switches, some of which are designed to be reversible; others have incorporated on switches, allowing for conditional activation only in the presensce of a specific agent [57]. Another approach that has been considered is T cell-depleting techniques such as antithymocyte globulin or alemtuzumab [88], or with cyclophosphamide [89, 90]. These agents are associated with increased infection risk and would be expected to eliminate CAR-T cells, and should only be considered in extreme circumstances.

Finally, extracorporeal adsorption of cytokines has been reported using Cytosorb®, which is a device designed for the treatment of cytokine storm and granted emergency approval for overwhelming systemic inflammation seen with COVID-19 infection. Cytosorb® has been reported in a single case in the setting of tocilizumab-refractory CRS with rapid response in clinical stability [91]. This has been used for severe refractory neurotoxicity as well [92]. A clinical trial is currently underway investigating the role of Cytosorb® for severe CRS (NCT04048434).

## Immune effector cell-associated neurotoxicity

The second most common potentially life-threatening toxicity seen with CAR-T cells is ICANS, a spectrum of neurotoxicy symptoms which encompasses encephalopathy, delirium, motor dysfunction including tremor, ataxia, dysphagia, and seizure. The ASTCT consensus group has defined this broadly as “a disorder characterized by a pathologic process involving the central nervous system following any immune therapy that results in the activation or engagement of endogenous or infused T cells and/or other immune effector cells. Symptoms or signs can be progressive and may include aphasia, altered level of consciousness, impairment of cognitive skills, motor weakness, seizures, and cerebral edema” [7].

ICANS was initially scored using CTCAE v4, but over time new grading systems have evolved to more specifically capture symptoms attributed specifically to CAR-T. The main challenge with grading IEC-related neurotoxicity is subjectivity regarding neurologic symptoms, especially encephalopathy, and so the CARTOX assessment was devised, then modified with incorporation of parts of the Mini-Mental State Examination into the CARTOX-10 encephalopathy assessment tool [27]. The CARTOX criteria include assessment of elevated ICP into grading, which can be difficult to measure as well as altered by multiple clinical variables, and so an ASTCT consensus group devised a further refined encephalopathy-scoring tool called the Immune Effector Cell-Associated Encephalopathy (ICE) score. ICE scores assess patients based on orientation, naming, command following, writing, and attention with points for each domain adding up to a maximum of 10 [7]. The ICE score is then added to assessments of additional domains of neurologic status: depressed consciousness, seizure, motor dysfunction, and cerebral edema. ICANS grading is summarized as follows [7, 8]:

- Grade 1: ICE score >6 with preserved alertness
- Grade 2: ICE score 3-6, mild somnolence but awakens to voice
- Grade 3: ICE score 0-2, somnolence responsive to tactile stimulation, brief seizure responsive to intervention, and/or limited cerebral edema on imaging
- Grade 4: ICE score 0, profound somnolence, life-threatening prolonged seizure or status epilepticus, diffuse cerebral edema, and/or symptomatic intracranial hypertension
- Grade 5: death

In patients youngter than 12 years or with developmental delay the ICE score is replaced by the Cornell Assessment of Pediatric Delerium [8, 93].

ICANS incidence varies with the specific CAR-T product and disease type. The ZUMA-1 trial using axi-cel for relapsed LBCL found ICANS to occur in 64% of patients, with severe in 44% of those cases. The JULIET trial investigating tisa-cel for LBCL had lower rates of ICANS with 26% of patients having any grade of ICANS and 58% of those being severe [11, 12]. The ZUMA-2 and -3 trials investigating KTE-X19 found similar rates of ICANS at 63 and 60%, respectively, with approximately half of each being grade >=3 [13, 14]. An important feature of ICANS is that it has been found to occur at higher rates when using a CAR-T products containing the CD28 co-stimulatory domain, which is apparent when compairing tisa-cel to axi-cel and KTE-X19.

ICANS tends to occur later after infusion than CRS. For example, on the JULIET trial the median time of onset for CRS was 3 days, compared to 6 days for ICANS [12]. Similar timing is observed with axi-cel, where median onset of CRS on the ZUMA-1 trial was 2 days compared to 5 days for ICANS, and on the ZUMA-5 trial using axi-cel for non-Hodgkins lymphoma median onset of CRS was 4 days compared to 8 days for ICANS [11, 16]. Important risk factors for severe ICANS include high pre-infusion disease burden, history of neurologic disease, and development of severe CRS [25, 94, 95].

The biology of ICANS is not fully understood but does appear to be distinct from that of CRS despite both occurring following CAR-T administration. Investigations into the biological correlates of ICANS have identified serum levels of IL-6, IL-18, IFNγ, IL-8, IL-10, and TGFβ to be associated with severe toxicity [96], with increased IL-18 recently identified as a correlate [22]. In the CSF, it was found that concentrations of WBC and CAR-T cells alone did not correlate with neurotoxicity, however levels of IL-6, IL-8, MCP1, and IP10 did correspond [25]. Separately, elevations in the terminal complement complex soluble C5b-9 was found to be significantly associated with ICANS [22], which supports a role for vascular endothelial dysfunction causing blood brain barrier (BBB) disruption as suggested by others [25, 26]. Additionally, excitatory neurotransmitters glutamate and quinolinic acid were elevated in the CSF of patients with ICANS suggesting a mechanistic link to symptoms such as seizures and tremor [25].

Treatment regimens for ICANS (Table 2) are less well established than those for CRS. Tocilizumab is not effective for the treatment of ICANS [11, 25, 104], possibly because tocilizumab, similar to other monoclonal antibodies, does not readily cross the BBB [105]. CS are the primary therapy for ICANS, with expert opinion supporting the use of dexamethasone in particular due to CNS penetration, or methylprednisolone [8, 50, 90]. The ZUMA-1 and -3 trials found reduced incidence of severe ICANS with early CS initiation [14, 76]. For persistent neurotoxicity, dexamethasone is often switched to high-dose methylprednisolone. Increasing CS dosages raises the concern for causing CAR-T dysfunction, as well as significant side effects including metabolic derangements, hypertension, and infection.

**Table 2:** Established and emerging therapies for ICANS

	Agent	Rationale	Comments	Notable references
First line	Corticosteroids	Global immunosuppression with CS currently has the most evidence of efficacy; antibody-based therapies such as tocilizumab do not cross the BBB.	Dexamethasone preferred due to CNS penetration, however package insert for FDA-approved CD19 CART recommends dexamethasone or methylprednisolone. High-dose methylprednisolone recommended for severe toxicity. Prophylactic steroid administration found to be effective in preventing higher grade CRS and ICANS	[8, 11, 76]
Second line	Anakinra	IL-1 plays a major role in CAR-T-mediated toxicity. Anakinra crosses the BBB.	Increasingly being used for ICANS, however steroids still considered first line. Increasing interest in utility for prophylaxis of toxicities with evidence particularly for ICANS mitigation.	[25, 55, 97, 98]
Additional therapies	Intrathecal corticosteroids +/- chemotherapy	Decrease CNS inflammation directly, no interference from the BBB	LP may be challenging in severely ill patients, often who have thrombocytopenia.	[99, 100]
	Intrathecal chemotherapy (MTX, Ara-C)	Ablate CAR-T cells in the CNS, no interference from the BBB	Effective for refractory ICANS in a small number of patients. Likely to destroy CAR-T cells, at least in CNS	[101]
	ATG	Direct elimination of T cells to abrogate CAR-T toxicity	Single case reported. Indiscriminate T cell targeting would be expected to eliminate CAR-T, however long-term persistence reported. Significant infection risk associated with ATG	[102]
	Defibrotide	Stabilization of the endothelium, which is disrupted in ICANS	Phase 2 trial ended early for lack of efficacy using prophylactic defibrotide	[103]

Abbreviations: *MTX* methotrexate, *Ara-C* cytarabine, *BBB* blood brain barrier, *CNS* central nervous system, *LP* lumbar puncture

Recent practice in the treatment of ICANS reflects the finding that IL-1 plays a significant role in CRS and ICANS [77]. Anakinra is increasingly being used successfully, particularly for ICANS refractory to first-line CS in both adult and pediatric populations [55, 97, 106], and when started early may allow for a more rapid taper of CS [98]. Given these experiences, anakinra is recommended as a second-line agent for ICANS [107], and is being investigated as a prophylactic agent to reduce or prevent toxicity with interim analyses reporting promising reductions in ICANS frequency and severity [71].

Numerous therapies for subsequent-line treatment of refractory ICANS have been proposed. Considering the challenge posed by the BBB, a few patients have been treated with intrathecal hydrocortisone with or without chemotherapy for systemic steroid-refractory disease. Shah et al report rapid and sustained resolution of severe ICANS with IT hydrocortisone and methotrexate with or without cytarabine in two patients [99], and others have demonstrated success with only IT hydrocortisone with or without chemotherapy, finding an overall reduction in total CS dose [100]. Wang et al. describe a patient who developed severe grade 4 cerebral edema after KTE-X19 who was treated with antithymocyte globulin (ATG) and found a rapid decline in CAR-T cell numbers in peripheral blood as well as IL-2 and IFNγ serum concentrations followed by clinical resolution of neurotoxicity within a week [102]. Surprisingly, the authors note that there was lower level CAR-T persistence followed by complete remission over the 24 months reported. Considering the potential role of endothelial dysregulation in ICANS, defibrotide has been investigated as an agent for stabilizing the endothelium. While initial results projected a significant reduction in ICANS rate, a phase 2 trial using defibrotide in a prophylactic role found more modest reductions in ICANS rate and duration, and the trial was terminated early for lack of efficacy [103].

There have now been several cases of a distinct syndrome of bradykinesia associated with limb rigidity, tremor, and other symptoms seen in Parkinson’s disease [108]. To date, all of these cases have occurred after treatment with CAR-T targeting BCMA, and have occurred weeks to months following infusion [32]. Concerningly, many of these cases have proven to be irreversible, although more recently two patients had recovery of symptoms after treatment with CS, anakinra, and high-dose cyclophosphamide [109]. Symptoms appear to correlate with CAR-T infiltration of the CNS, specifically the basal ganglia, suggesting this may be an on-target off-toxicity effect specific to BCMA [43].

## Immune effector cell-associated hlh-like syndrome

Some patients with severe CRS develop a state of hyperinflammation that is accompanied by hyperferritinemia, cytopenias, hypofibrinogenemia, and multiorgan dysfunction. It was recognized early on that this was analogous clinically and biochemically with HLH [3, 20], and over time it was observed that some patients developed a similar HLH-like state either after initial improvement from CRS or after complete resolution of CRS. This state does not typically respond to tocilizumab in the same way as severe CRS and is associated with worse outcomes [110–112]. As greater numbers of patients with CAR-T-related toxicities were observed, this syndrome has been defined as an entity distinct from severe CRS based on its time course as it suggests a separate underlying biology.

HLH is defined as a disorder characterized fever, hepatosplenomegaly, organ failure, neurologic toxicities, coagulopathy, cytopenias, and hyperferritinemia, and is caused by immune activation due to dysregulated interactions between cytotoxic T cells, macrophages, and natural killer cells resulting in a cascade of cell- and cytokine-mediated tissue damage [113, 114]. HLH is separated into primary HLH (pHLH), caused by germline mutations in immune regulation, classically involving dysfunction in CD8 T cell or NK cells resulting in unchecked activation of macrophages, and secondary HLH (sHLH) characterized by HLH after an initial dysregulating disease such as an autoimmune disorder, EBV infection, malignancy, metabolic disorders, or overwhelming infection [115, 116]. A multidisciplinary team of experts recently defined the syndrome of HLH-like disease following immunotherapy as “Immune effector cell associated HLH-like syndrome,” or IEC-HS [6]. The incidence of IEC-HS has been reported to be ~3.5% across different CAR-T constructs and targets in adults, but varies with CAR-T construct, just as the persistence and propensity to cause CRS/ICANS varies between construct. As an example, a CD22-targeting CAR-T product using a 4-1BB costimuatory domain resulted in a HLH-like syndrome in 21 of 59 patients treated on one trial, much higher rates than previously reported following CART19 [117]. Unlike CRS, IEC-HS is associated with a poor prognosis [79]. Additionally, while the severity of IEC-HS has not been found to correlate with CRS severity, to date all cases of IEC-HS have occurred after or with resolving CRS [6].

The grading of IEC-HS has not been formally established, however the ASTCT consensus group outlines a grading scheme that will likely continue to evolve prior to being universally adopted. The general outlines of the IEC-HS grading system are as follows and adapted from expert panels [6, 90]:

•Grade 1: mild symptoms including fever, but clinical stability not requiring intervention

•Grade 2: mild to moderate symptoms such as hypotension responsive to fluids alone and/or hypoxia requiring low-flow nasal cannula, asymptomatic hypofibrinogenemia

•Grade 3: More severe symptoms including hypotension responsive to a vasopressor, respiratory distress requiring non-invasive support, coagulopathy with bleeding symptoms

•Grade 4: Severe, life-threatening toxicities including respiratory distress requiring intubation, hypotension requiring multiple vasopressors, and/or dialysis

•Grade 5: death

**Fig. 2:**
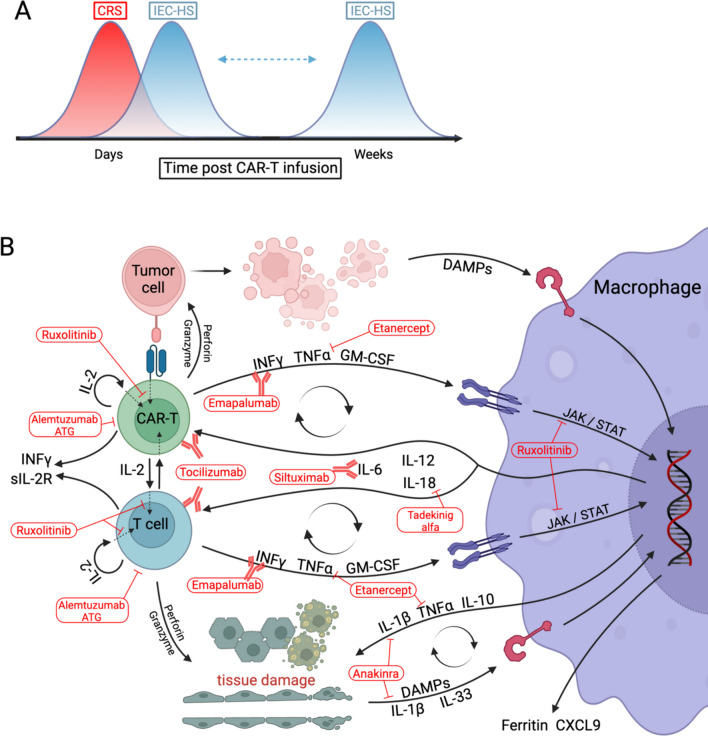
IEC-HS timing is disctinct from that of CRS in that it occurs as CRS is resolving or later, after CRS has resolved (**A**). Schematic detailing the current understanding of the pathophysiology of IEC-HS, inferred from lines of evidence in sHLH as well. The general concept is that CAR-T activation creates feed-forward loops with macrophages and endogenous T cells, activated by and resulting in cytokine secretion and tissue damage. The mechanisms of action of several proposed therapies for IEC-HS are incorporated into the schematic to illustrate how they may work to ameliorate this pathology (**B**). Created with biorender.com

A distinctive feature of both severe CRS and IEC-HS is a very high ferritin, with values frequently greater than 10,000 ng/mL, a value that is considered sensitive and specific for MAS/HLH [20]. While some have proposed using a ferritin value of 10,000 ng/mL as diagnostic criteria [90], the ASTCT definition is more inclusive and delineates a level of twice the upper limit of normal, twice the ferritin for that patient at time of infusion, or rapidly rising ferritin, and note that while experience is insufficient to define a threshold level, a normal ferritin has a high negative predicative value. Additional diagnostic elements from the ASTCT are “features of macrophage activation/HLH, attributable to IEC therapy, and associated with progression or new onset of cytopenias, hyperferritinemia, coagulopathy with hypofibrinogenemia, and/or transaminitis.” There is also an emphasis placed on delayed onset, as this often manifests as CRS is resolving or has completely resolved [Fig. 2a]. Unlike CRS, fever is not required for the diagnosis of IEC-HS [6].

While specific cytokine levels were not included in the recent ASTCT diagnostic criteria, cytokines likely play a substantial role in IEC-HS. IL-1b has been shown to be upregulated in patients with HLH-like disese after CAR-T [117] and anakinra has been shown to be an effective treatment for HLH/MAS [118, 119]. IFNγ is elevated in primary and secondary HLH [120–122], and essential for disease development in an animal model [123]. Emapalumab is approved for the treatment of pHLH [124, 125]. A model of the biology of IEC-HS can be proposed in which macrophages are activated by CAR-T via IFNγ signaling, as well as by damage signals from target cells being killed by CAR-T. Activated macrophages in turn recruit effector cells and more macrophages/monocytes and activate effectors (including endogenous CD8 T cells) via stimulatory cytokines such as IL-12, IL-18, and IL-6. This creates a feed forward loop resulting in widescale macrophage activation and proliferation causing collateral tissue damage, accompanied by high levels of ferritin and secretion of high levels of IL-1b and TNFα which further mediate tissue damage and immune cell activation (Fig. 2b). IFNγ blockade with emapalumab is approved for pHLH [126] and is being studied in MAS based on favorable outcomes in a pilot study of a small number of patients with refractory disease [127]. IFNγ elevation in IEC-HS has been reported [59, 79, 117]. The efficacy of emapalumab was demonstrated by Rainone et al. who described a patient with relapsed/refractory Ph-like B-ALL treated with tisa-cel who had CRS that improved within the first week after infusion, then developed persistent fevers, transaminitis, and ferritin levels that increased from 3,000 to 15,000 ng/mL over 24h before rising above the detectable range along with development of hemodynamic instability, coagulopathy, worsening cytopenias, hypertriglyceridemia, and hyperbilirubinemia, as well as a high IFNγ serum concentration despite repeated dosing of tocilizumab. The patient was initially treated with CS and anakinra with some clnical improvement, then within a few hours of receiving emapalumab the patient defervesced and stabilized, and anakinra and steroids were able to be weaned off over the subsequent 6 days [24]. CXCL9 is a useful biomarker for monitoring response to emapalumab [126, 128].

Historically, CS were first line for sHLH, however with the advent of newer targeted therapies it is attractive to limit CS use due to their side effects. Suggested therapies for IEC-HS include anakinra, often with CS, based on the established roles of both agents in sHLH. Given more recent description of this entity development of a consensus definition treatment guidelines for IEC-HS are evolving and several options have been used or proposed (Table 3). Growing evidence and experience supports the use of ruxolitinib, the Jak1/Jak2 inhibitor, capitalizing on the JAK/STAT signaling pathway being a common route for pro-inflammatory signals to exert their effects [136]. Ruxolitinib is used increasingly for frontline or salvage therapy in HLH [137, 138], and has been successfully used recently for refractory IEC-HS [112]. The ASTCT consensus group has ultimately advised consideration of ruxolitinib as second line for IEC-HS.

**Table 3:** Proposed therapies for IEC-HS

	Agent	Rationale	Comments	Notable references
First Line	Corticosteroid	Widely acting immunosuppressive effects, historically first-line (in combination) for pHLH and sHLH	CAR-T cell compromise continues to be a concern. Side effects include infection risk, hypertension, metabolic derangements	[113]
	Anakinra	IL-1b is upregulated in IEC-HS, often used first-line with CS in MAS	Successful use in IEC-HS. Good side effect profile, can be titrated to effect	[117]
Second line	Ruxolitinib	Blocks signaling through multiple cytokine receptors	Successful use in refractory IEC-HS. Risk of worsening cytopenias and viral reactivation	[112]
	Emapalumab	IFNγ is elevated in primary and secondary HLH, animal models show essential role for IFNγ in HLH	Successful use in CAR-T toxicity and in small pediatric cohort. Evidence supports that emapalumab does not impede CAR-T efficacy	[24, 79, 117]
Additional and emerging therapies	Tocilizumab/ Siltuxumab	IL-6 blockade effective in CRS, all cases of IEC-HS have followed or accompanied CRS	Use discouraged in absence of CRS, may have a role in preventing severe toxicities such as IEC-HS when used pre-emptively	[129]
	Etoposide	Topoisomerase inhibitor that induces apoptosis in proliferating T cells	Relatively extensive use in pHLH and sHLH, and has been used successfully in refractory IEC-HS. Proposed as second-line therapy by some, however is a cytotoxic agent with nontrivial side effect profile and risk for secondary malignancy.	[8, 90]
	Alemtuzumab	CD52 is present on mature lymphocytes including T lymphocytes used in the production of CAR-T	Has been used in primary HLH, in particular for refractory disease. Increased risk for infectious complications and very hard to obtain in United States	[130], [131]
	ATG	Horse or rabbit-derived antibodies against T lymphocytes and thymocytes to target CAR-T cells	Limited experience in HLH, increased risk for infectious complications	[132]
	Canakinumab	IL-1b is upregulated in IEC-HS, often first line with CS in MAS	Limited experience with HLH, has been used for refractory MAS/HLH; antibody therapy less likely to cross blood brain barrier	[133]
	Tadekinig alfa	IL-18 elevated in patient with HLH and MAS, is a potent inflammatory cytokine and enhances IFNg secretion	Interest based on limited experience in XIAP deficiency causing pHLH	[134]
	Etanercept/ Infliximab	TNFα is elevated in HLH and mediates systemic damage	Clinical experience limited	[135]

Additional options have been suggested for IEC-HS. IL-6-directed therapy with tocilizubmab or siltuxumab may be beneficial, although this could primarily apply only to IEC-HS with concomitant CRS. Targeted destruction of T cells with ATG and alemtuzumab is used in HLH [130, 132, 139], however such a stragety would be reserved for only the most refractory cases of IEC-HS. More targeted approaches of interest, largely theoretical at the time of this publication, include IL-18 blockade with tadakinig alfa which has limited experience in a subtype of pHLH [134], and is the subject of a trial for MAS therapy (NCT03512314), and TNFα blockade owing to the observation that this cytokine is frequently elevated in HLH and IEC-HS as well [135, 141, 140].

Based on experience in pHLH, the topoisomerase inhibitor etoposide has been used in IEC-HS, and is considered by some to be second-line [8, 90]. Etoposide abrogates effector cell activity through induction of apoptosis rather than more inflammatory cell death mechanisms like pyroptosis [142, 143]. However, avoidance of effector cell destruction is of course beneficial if possible, and etoposide causes increased risk of secondary malignancies. We do not recommend the use of etoposide outside of exceptional circumstances.

Ultimately, both early severe CRS and the later IEC-HS have clinical, biologic and pathophysiologic features highly overlapping with HLH, including high ferritin, low fibrinogen, multisystem organ failure, cytopenias, and hypercytokinemia with elevated IFNγ, IL6, and IL10. IEC-HS portends a worse prognosis, and is less likely to respond to therapies including tocilizumab. Future work is needed to unravel the mechanistic differences between these two entities to inform treatment, especially in refractory cases.

## Conclusions

The field of CAR-T cell therapy has grown rapidly in the last 10 years, and our understanding of CAR-T associated toxicities has evolved with it. The definition of CRS has been refined, and now management recommendations can be definitively made through three lines of therapies, with several additional agents showing promise in refractory cases and preemptive strategies emerging as well. ICANS remains pooly understood biologically, although some progress has been made. While a better understanding of ICANS would be helpful for developing treatment strategies, advances in treatment have been made recently and IL-1 blockade with anakinra has emerged as a potent therapy. IEC-HS is a newly-recognized, and named, entity describing a later onset cytokine storm with organ dysfunction following CAR-T. A key feature of IEC-HS is timing: IEC-HS occurs as CRS is resolving or after CRS has resolved. However, it is not clear that this is a biological imperative and CRS and IEC-HS may not be mutually exclusive. Further elucidation of the biological mechanism of each process may help define criteria to better distinguish the two.

Future work should focus on comparative trials with patients randomized to alternative toxicity management strategies to more directly evaluate options and guide clinical decisionmaking. Determination of definitive recommendations is certain to be challenged by the expanding application of CAR-T cell products to more diseases. As new CAR-T products are developed with different features and targets specific immune toxicities may differ. A deeper understaning of the pathophysiology of CRS, ICANS, and IEC-HS will be crucial to achieve continued progress in toxicity amelioration and prevention.

## Data Availability

No datasets were generated or analyzed in the writing of this manuscript.
